# 

**Published:** 1987-02

**Authors:** 


NO.1 1987

--------SEQ: B33740000

BRISTOL MEDICO-CHIRURGICAL

JOURNAL

### BRISTOL MEDICO-CHIRURGICAL JOURNAL

VOLUME 102(i) FEBRUARY 1987

NATIONAL
LIBRARY
JUN 22 1987
OF
ME[illegible]CINE

Should every cow carry a government health warning?
Bogus Allergy Treatment
Frenchay and Ham Green Hospitals
Woodcarving
Our correspondents in Paris and Singapore

IN ASSOCIATION WITH

BRISTOL MEDICINE

THE MEDICAL JOURNAL OF THE SOUTH WEST

